# A systematic review of endometrial cancer clinical research in Africa

**DOI:** 10.1186/s13027-023-00563-2

**Published:** 2024-01-12

**Authors:** Chidinma P. Anakwenze, Agnes Ewongwo, Louisa Onyewadume, Ademola Oyekan, Chinelo Onwualu Chigbo, Luca Valle, Yimin Geng, Paul Olapade, Kenechukwu Okwunze, Nwamaka Lasebikan, Anuja Jhingran, Onyinye D. Balogun, Atara Ntekim

**Affiliations:** 1 Department of Radiation Oncology, MD Anderson Cancer Center, Houston, TX USA; 2 Department of Radiation Oncology, Weill Cornell Medicine, New York, NY USA; 3 Department of Radiation and Clinical Oncology, University of Nigeria Teaching Hospital, Enugu, Nigeria; 4 Department of Radiation Oncology, Stanford University, Palo Alto, CA USA; 5 Research Medical Library, MD Anderson Cancer Center, Houston, TX USA; 6 Department of Radiation Oncology, College of Medicine, University of Ibadan, Ibadan, Oyo State Nigeria; 7 College of Medicine, University of Ibadan, Ibadan, Oyo State Nigeria; 8 Harvard University School of Public Health, Boston, MA USA; 9 Department of Radiation Oncology, University of California Los Angeles, Los Angeles, CA USA

**Keywords:** Endometrial cancer, Africa, Systematic review

## Abstract

**Background:**

Women in Africa are experiencing a rising burden of endometrial cancer. Research and investment to improve treatment and outcomes are critically needed. We systematically reviewed and characterized endometrial cancer-related research within a clinically relevant context to help organize and assess existing endometrial cancer research in Africa.

**Methods:**

According to PRISMA guidelines, we searched online databases for published endometrial cancer articles from African countries from January 1, 2011, to July 20, 2021. Based on our inclusion and exclusion criteria, independent reviewers documented the study design, country/region, human development index, focus of research, type of interventions performed, and histologic and molecular type to illustrate the breadth of research coverage in each region.

**Results:**

A total of 18 research articles were included. With an average Human Development Index (HDI) in Africa of 0.536, the average HDI of the represented countries in this study was 0.709. The majority (88.9%) of prospective endometrial cancer research articles in Africa were from North Africa, with Egypt encompassing 83.3% of the papers. Most of these studies focused on endometrial cancer diagnosis. Research on the treatment of endometrial cancer is still emerging (33% of papers). Of all included articles, only 11.1% represented Sub-Saharan Africa, where the majority population of black Africans reside.

**Conclusions:**

Endometrial cancer research in Africa is extremely limited, with the majority being concentrated in African countries with higher HDIs. As the incidence of endometrial cancer rises in Sub-Saharan Africa, there is a pressing need for more prospective clinical research to tackle the growing disease burden and improve outcomes.

**Supplementary Information:**

The online version contains supplementary material available at 10.1186/s13027-023-00563-2.

## Background

Endometrial cancer is the leading cause of gynecologic cancer mortality in high-income countries and is increasing in incidence in low- and middle-income countries, in part due to increasing rates of obesity, physical inactivity, and changes in child-bearing patterns. Between 1990 and 2017, there was a 75.7% increase in the total disability-adjusted life years (DALYs) due to endometrial cancer in sub–Saharan Africa [1]. The burden of endometrial cancer in Africa is projected to continue on an upward trajectory, as IARC estimates a twofold increase in both endometrial cancer incidence and mortality over the next two decades [2]. While the current distribution of incident endometrial cancer cases is similar across the regions in Africa, the situation is not as straightforward when assessing the context of its burden. The impact of the rising endometrial cancer burden is expected to be more severe in East and Southern Africa, accounting for 42.4% of Africa’s new endometrial cancer cases (11.5 out of 27.1 thousand) by 2040 despite only making up approximately one-third of the continent’s population as of 2019 [3, 4].

In the United States, where endometrial cancer is the most common gynecologic cancer, African American (AA) women experience an 80% higher mortality rate and a 22% difference in 5-year survival compared to Caucasian women [5, 6]. This disparity remains across stage and histologic subtypes, with studies showing a 2–3 times higher rate of more aggressive histologic subtypes (serous and clear cell adenocarcinoma as well as sarcomas) in AA women [5–9]. This histologic distribution is mirrored in sub-Saharan Africa, where 60% of endometrial cancer cases in one Nigerian cohort had poorly differentiated histology [10]. The causes of survival disparities across races are multifactorial, with differences attributed to socioeconomic, biological, and cultural factors. In Africa, where cancers are frequently diagnosed in advanced stages due to late presentation [11, 12], infrastructural challenges also result in diagnostic and treatment delays, further worsening survival outcomes [13, 14]. Differences in genetic makeup are another important contributor to survival disparities between races. Notably, of the 370 tumors included in the endometrial cancer molecular profiling by The Cancer Genome Atlas (TCGA), the majority were from Caucasian women, and few had the high-risk histology categories that appear in women of African descent [15, 16].

Although endometrial cancer is the third most common gynecological cancer in Africa, it is likely that this distribution will be altered over the coming decades to reflect the current situation in high-income countries [3]. This shift is anticipated due to an increasing adaptation of “western” lifestyles, including dietary and behavioral patterns. This growing disease burden highlights the need for endometrial cancer research in Africa to curb this trend and provide knowledge that will assist in prioritizing funding and directing efforts for prevention and control [17]. Several evidence-based initiatives have recently been employed to improve the standard of care for cancer patients in Africa. For instance, in Botswana, healthcare professionals and trainees in two oncology centers participate in monthly virtual tumor boards under the BOTSOGO collaboration with Massachusetts General Hospital [18]. Despite these advances, given the growing endometrial cancer burden in Africa and the paucity of prospectively collected data or endometrial cancer clinical trials, there is still a need for more research to guide evidence-based strategies in Africa [19]. We thus aim to describe the current landscape of endometrial cancer clinical research in Africa, which may help identify gaps and serve as support for future studies. We will also describe the histologic distribution of endometrial cancer in African countries.

## Methods

According to Preferred Reporting Items for Systematic Reviews and Meta-analysis (PRISMA) guidelines [20], we conducted a systematic literature search of Ovid MEDLINE, Ovid EMBASE, Clarivate Analytics Web of Science, Wiley-Blackwell Cochrane Library, and WHO African Index Medicus database for publications in all languages from January 1, 2011, to July 20, 2021. This study was institutional review board-exempt given that it is a systematic review. The concepts searched included “*endometrial neoplasms*”, *“endometrial cancer*”, “*Africa*” and *“African countries*”. Both subject headings and keywords were utilized. The list of African countries was based on the United Nations and African Union member states [21, 22]. All languages were included. The complete search strategies are detailed in Additional file 1: Appendix Tables S1–S5.

Inclusion criteria included experimental studies (i.e., clinical trials), observational studies (prospective cohort and cross-sectional studies) and retrospective studies conducted in Africa that focus on the management of endometrial cancer. There was no restriction based on the language of publication. Exclusion criteria included animal or nonhuman studies, in vitro studies, studies only available as meeting abstracts, review papers, editorials, commentaries, reports, pathology studies, case reports, and studies on screening and diagnosis of endometrial cancer.

Two independent reviewers examined the titles and abstracts of selected articles and assessed studies for inclusion using the inclusion and exclusion criteria above. The full text was reviewed for abstracts without sufficient information or in the case of a disagreement. Covidence software was used to screen studies, report data, and document study quality. For abstracts that passed the initial screening, the full text was retrieved for secondary screening. For articles that were not easily accessible, we contacted study authors and/or requested the article via interlibrary loan. In cases where we were unable to obtain the full texts, the articles were excluded. The full texts of the selected studies were reviewed independently by two reviewers to confirm eligibility. A study was included when both reviewers independently assessed it as satisfying the selection criteria after review of the full text. A third reviewer mediated in the event of disagreement following discussion. Reasons for exclusion were recorded.

Data extraction and quality assessment were performed in duplicate by two independent reviewers with discordances resolved by a third reviewer. We used a spreadsheet to collect information regarding title, first author, journal, year of publication, country, study design, study setting, and type of interventions performed. We assessed whether the study included stage at diagnosis, survival probability outcomes or both. We recorded the number of included patients, year of diagnosis, age at diagnosis, other reported demographic characteristics, histologic and molecular type, and tumor grade (Table 1). Quality assessment results are presented in Additional file 1: Appendix Tables S6–S7.

**Table 1:** Study characteristics

Country region^a^	North (n, %)	South (n, %)	Total (n)
No. of studies	16 (88.89%)	2 (11.11%)	18
Human Development Index [40]			
Low (< 0.550)	–	–	–
Middle (0.550–0.699)	–	–	–
High (0.700–0.799)	16 (88.89%)	2 (11.11%)	18
Very high (≥ 0.800)	–	–	–
Study design			
Case control	–	1 (5.56%)	1
Cohort	11 (61.11%)	–	11
Cross-sectional	2 (11.11%)	–	2
Cross-sectional/diagnostic accuracy^b^	–	1 (5.56%)	1
Nonrandomized experimental	1 (5.56%)	–	1
Randomized controlled	2 (11.11%)	–	2
Funded			
Yes	2 (11.11%)	1 (5.56%)	3
No	6 (33.33%)	–	6
Not specified	8 (44.4%)^c^	1 (5.56%)	9
No. of centers			
Single	15 (83.33%)	2 (11.11%)	17
Multiple	1 (5.56%)^d^	-	1
Patient population			
Oncology institute	1 (5.56%)^c^	–	1
University	15 (83.33%)	1 (5.56%)	16
Not specified (in urban area)	–	1 (5.56%)^d^	1
Year of study publication			
2010–2013 (included)	5 (27.7%)	–	5
2015–2018	4 (22.2%)	–	4
2019–2021	7(38.9%)	2 (11.11%)	9
Conflict of interest			
None	14 (87.5%)^c^	1 (5.56%)	16
Not specified	2 (11.11%)	1 (5.56%)	3
Funded			
Yes	2 (11.11%)	1 (5.56%)	3
No	6 (33.3%)	–	6
Not specified	8 (44.4%)^c^	1 (5.56%)	9
Histology (%)^f^			
Endometroid adenocarcinoma	12 (66.67%)	1 (5.56%)	12
Serous/papillary serous carcinoma	6 (33.3%)^c^	–	6
Clear cell carcinoma	2 (11.11%)	–	2
Carcinosarcoma	1 (5.56%)^c^	–	1
Uterine sarcoma	–	–	0
Unknown	2 (11.11%)	1 (5.56%)	3
Mean age at diagnosis (years)			
< 60	8 (44.4%)	–	8
** ≥ **60	4 (22.2%)	1 (5.56%)	5
Unknown	4 (22.2%)	1 (5.56%)	5

^a^There were no data from the east and west regions, so they were not included in this table

^b^This study is 1 of 2 South African studies from the same patient population and same author (the study that described the population as a cohort was excluded)

^c^Includes 1 Tunisian study

^d^Includes 1 Egyptian study

^e^Includes 1 South African study

^f^Many articles addressed more than one histology. Other histologies not included in the table include adenosquamous, nonendometroid, and mixed endometrioid adenocarcinoma

**Fig. 1:**
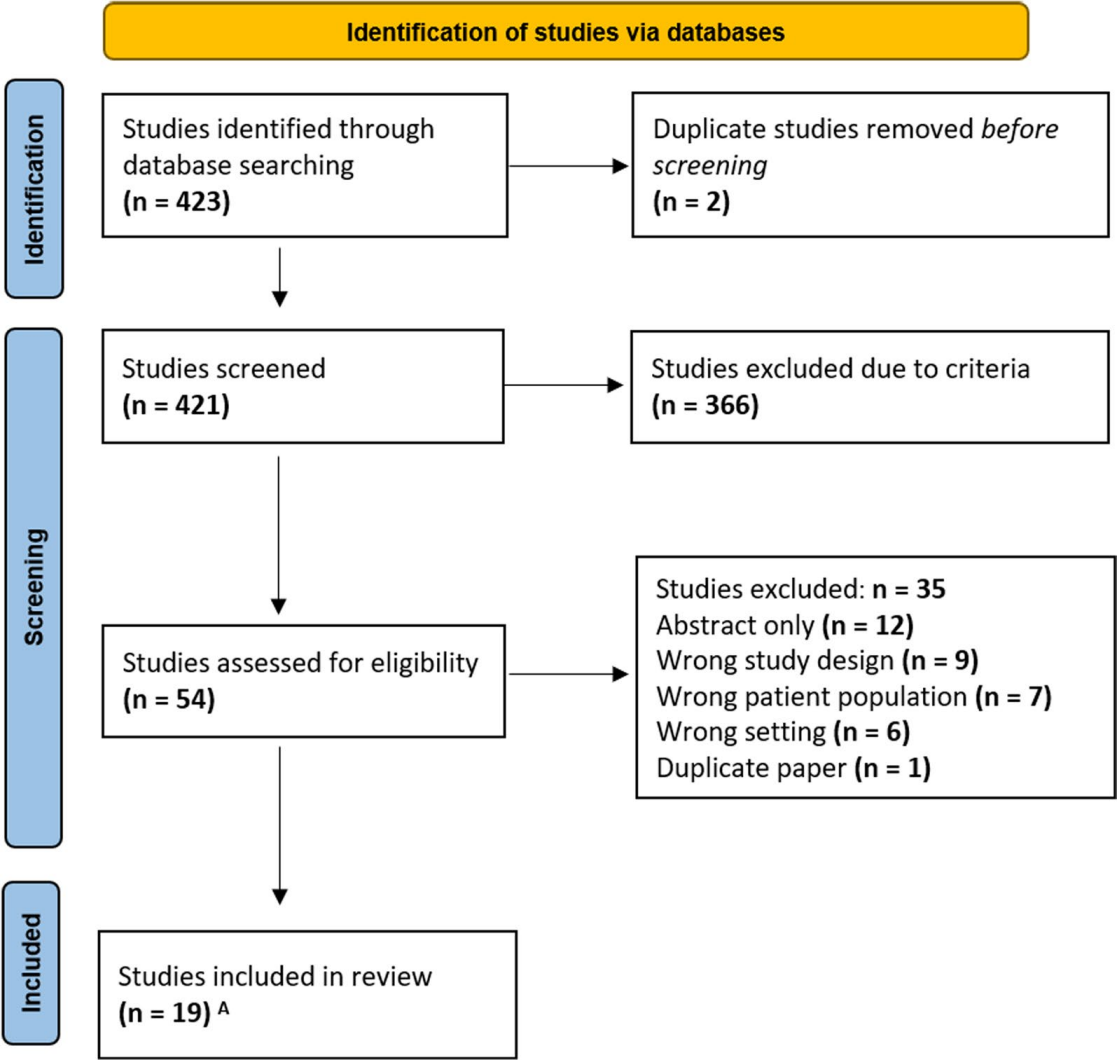
PRISMA flow diagram of the number of searches yielded, excluded, and reviewed. ^A^Includes 2 South African studies from the same patient population and same author (1 study was excluded during further analysis)

Data were reported in narrative and statistical form using figures, tables, and graphs. A PRISMA flowchart was created (Fig. 1). We reported the study design, country/region, human development index, focus of research, type of interventions performed, and histologic and molecular type to illustrate the breadth of research coverage in each region. We described the number and types of articles included. The Human Development Index was used to group countries for subgroup analyses. The Newcastle‒Ottawa Quality Assessment Scales [23] for the cohort and case control studies were used to assess the risk of bias. A modified Newcastle‒Ottawa scale [23] was used for bias assessment of the cross-sectional studies, and the Cochrane Risk Of Bias 2 (ROB2) scale [24] was utilized for assessing bias in the randomized control trials. These involved assessment of bias risk in each of the following three categories: selection, compatibility, and outcome (see Table 2). Two independent reviewers reviewed the studies for risk of bias, and potential dependencies were resolved by consultation with a third researcher.

**Table 2:** Results of critical appraisal of included randomized controlled trials using Cochrane Risk Of Bias 2

Study	Study design	Total bias risk
El-Agwany, 2018	Randomized control	High
Fayallah, 2011	Randomized control	Some concern

## Results

A total of 18 research articles comprising 991 patients were included in this review. Although 19 studies (with a total of 1136 patients) met the inclusion and exclusion criteria, all aggregate values and percentages were based on 18 studies (i.e., one was excluded). This was because 2 studies that were performed by the same lead author utilized the same patient population, which they alternately described as a cohort versus a cross-sectional/diagnostic accuracy study.

**Fig. 2:**
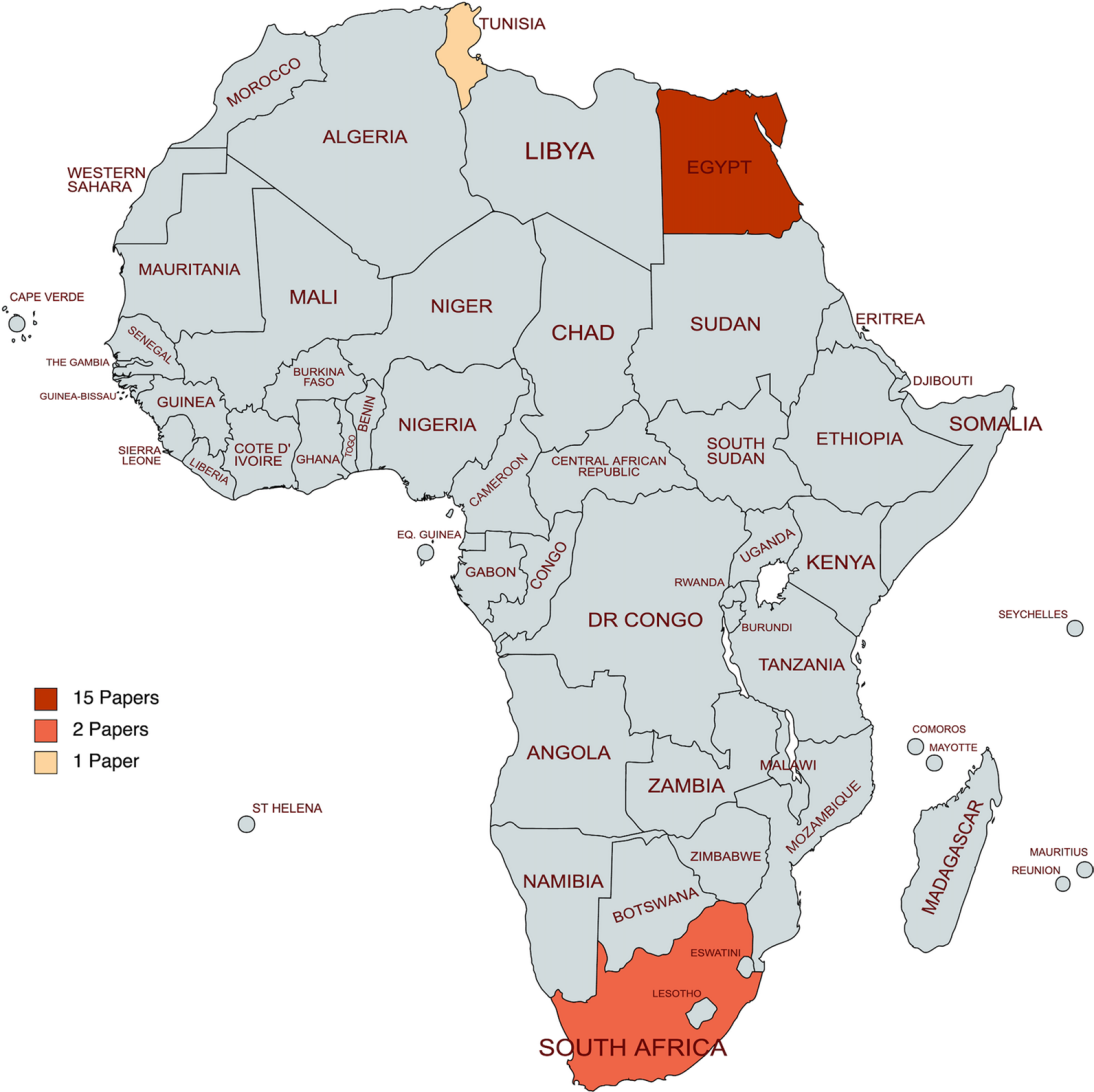
Geographic Distribution of Prospective Endometrial Cancer Research within Africa. The map represents individual countries only and does not clearly illustrate some of the smaller African countries

As illustrated in Fig. 2, the majority of papers were from Egypt, followed by South Africa. The majority (88.89%) of prospective endometrial cancer research in Africa was from North Africa, with Egypt encompassing 83.33% of all papers. Most of these studies focused on advanced imaging modalities. Research on the treatment of endometrial cancer is still emerging, with only one-third of the reviewed publications addressing it and 67% being diagnostic related. Of all the included articles, only 11.11% represented Sub-Saharan Africa, all from South Africa. While the average Human Development Index (HDI) in Africa is 0.536 [25], the average HDI of the represented countries in this study was 0.709 (min 0.707, max 0.740). The three countries represented, Egypt, South Africa, and Tunisia, all had high HDIs of 0.707, 0.709, and 0.740, respectively.

There has been an increase in the number of studies published recently, with 50.01% of papers having been published from 2019 to 2021 compared with 27.7% of papers from 2010 to 2013 and 22.2% from 2015 to 2018. Although these studies were mostly designed as cohort studies (61.11%), cross-sectional studies and randomized controlled trials were the second- and third-most common study designs (both 11.1%). All but one study was performed at a single center (94.5%). Only 16.67% of studies had confirmed funding sources, 33.33% were unfunded and 49.96% had unknown funding. The majority (89.4%) of studies were performed in the university setting. The remaining population was equally divided between an oncology institute setting (5.56%) and the urban setting of Soweto (5.56%).

There were a total of 991 patients in these studies. For studies that reported age of diagnosis (*n* = *15*, 83.3%), there was no consensus method of reporting age, with 12 studies (66.7%) reporting age ranges for a cumulative range of 31–81 years old. Thirteen studies (72.2%) reported the mean age with an average of 57.97 years old (min 49.5, max 66.4) across all studies, and 4 studies (22.2%) reported the median age with an average of 59.25 years old (min 58, max 60) across all studies. Three out of 4 studies reporting median age had a median age < 60 years old. The majority of studies (n = 8, 44.4%) reported mean age at diagnosis to be < 60 years old compared with “mean age ≥ 60” and “unknown mean age” each at 27.7% (n = 5).

Although multiple articles included multiple histologies of endometrial cancer, most articles addressed endometroid adenocarcinoma (n = 13, 72.2%) and serous/papillary serous carcinoma (n = 6, 33.3%). Molecular classification was not well documented in all studies. Data on stage distribution were only reported in 7 studies (38.9%), and all these studies were from Egypt. Similarly, survival probability data were available for only 4 studies (22.2%), all from Egypt.

Critical appraisal of study quality & bias, performed using the appropriate bias tools for each study design (see Tables 2, 3 below), showed that apart from the randomized controlled trials, all other studies were scored as either “fair” or “good” quality when translated to AHRQ standards. Case‒control and cross-sectional studies with a range of 7–8 points were scored as “Good” studies, with each study attaining “3 or 4 stars in the selection domain AND 1 or 2 stars in the comparability domain AND 2 or 3 stars in the outcome/exposure domain” [23]. The cohort studies, with a range of 7–9 points, were scored as either “Good” (*n* = *8*) or “Fair” (*n* = *4*) studies, with the majority of studies attaining “3 or 4 stars in the selection domain AND 1 or 2 stars in the comparability domain AND 2 or 3 stars in the outcome/exposure domain” [23]. Cohort studies scored as “Fair”, either had deficiency in the selection or comparability domains. The two randomized controlled trials were scored as “High risk of bias” and “Some concerns”, respectively, due in large part to deficiencies in the “Outcome” and “Reporting” sections, suggesting a need for improvement of these sections during the study-planning phase.

**Table 3:** Results of critical appraisal of included observational studies using Newcastle‒Ottawa scores

Study	Study design	Total score
Ray, 2019	Case control	7, Good
Ghazala, 2021	Cohort	7, Fair
Abouhashem, 2016	Cohort	8, Good
Aly, 2013	Cohort	9, Good
El Sokkary, 2014	Cohort	8, Good
Gharib, 2020	Cohort	7, Good
Hamed, 2012	Cohort	8, Good
Mourad, 2017	Cohort	9, Good
Sanad, 2019	Cohort^a^	7, Fair^a^
Shady, 2016	Cohort	9, Good
Shatat, 2019	Cohort^a^	7, Fair^a^
Soliman, 2011	Cohort^a^	7, Fair^a^
Rady, 2019^b^	Nonrandomized experimental study	8, Good
Wadee, 2021^c^	Cross- sectional	8, Good
Elmahdy, 2019	Cross- sectional	7, Good
Ghorbel, 2020	Cross- sectional	7, Good

^a^These studies had an inadequate degree of control; thus, the total score was based on this

^b^This nonrandomized experimental study was evaluated as a cohort study

^c^This author utilized the same patient population for 2 studies, alternately describing the design as a cohort vs a cross-sectional/diagnostic accuracy study. The cohort study was excluded

## Discussion

The incidence rate of endometrial cancer has increased in several countries over successive generations, particularly in countries with rapid socioeconomic changes [26]. Given that over the past two decades, there has been a 75.7% increase in DALYs due to endometrial cancer in sub–Saharan Africa [1] and that the IARC has projected a twofold increase in its incidence and mortality over the next two decades [2], our systematic review is a timely attempt to define the state of endometrial cancer research from countries in Africa. We demonstrate that there is a dearth of data, with only 18 publications on this topic over the past 2 decades. Moreover, these data are concentrated in countries with high HDI and are mostly from North African nations, which has important implications for the generalizability of their findings to Sub-Saharan Africa.

In the United States, histology and socioeconomic factors have been shown to account for the difference in incidence, morbidity, and mortality between Caucasians and African Americans [27]. High-income countries often have different racial and ethnic variations in gynecologic cancers compared to low-to-middle-income countries [28]. As far back as 1992, Cronje et al. showed that preoperatively black women in Bloemfontein, South Africa were more likely to have advanced stages (II-IV) (*p* = *0.0024*) of endometrial adenocarcinoma per FIGO (Fédération Internationale de Gynécologie et d’Obstétrique) criteria and poorer tumor differentiation (*p* < *0.0001*) [29]. In addition, black women within those societies often have different genetic or hormonal factors contributing to the pathophysiology of their cancer [30]. Our systematic review showed that age at diagnosis was notably < 60 years old in the majority of recorded cases. Although this was unexpected and may be explained by the lower life expectancy in African countries, it also has important implications for diagnostic considerations in these settings.

As shown in low-income areas in the United States, patients from high-income settings have more access to research funding, improved treatment facilities, cutting-edge research trials, enhanced transportation for radiation, and improved monitoring of toxicities [31]. The ramifications for treatment options, including chemo- and immunotherapy, radiation therapy, and surgical resection, are innumerable; hence, marked improvement in outcome measures such as 5- and 10-year mortality in low- to middle-income countries may be difficult to achieve. The scarcity of research on endometrial cancer in Africa has resulted in a stagnation of the development of regional, evidence-based treatment guidelines. This deficiency has also impeded the build-up of relevant healthcare infrastructure and hindered the allocation of funding for both endometrial cancer treatment and prevention initiatives in the region. Addressing these research gaps is crucial for advancing comprehensive and effective strategies in the fight against endometrial cancer in Africa. As such, more needs to be done to invest in building research capacity in the form of infrastructure and research personnel in low-to-middle income countries.

Our systematic review showed that approximately two-thirds of the studies addressed diagnosis-associated issues, while one-third were treatment-related. Of these studies, only 2 (11.1%) were randomized controlled trials, whereas the rest were retrospective case‒control, cohort, or cross-sectional studies. In Western countries, a variety of research designs have been used to assess the use of biomarker-driven targeted therapy, adjuvant pelvic radiotherapy, lymphadenectomy, and hysterectomy approaches (i.e., laparoscopy vs laparotomy) for the management of endometrial cancer [32–38]. This diversity in clinical trial options is also needed in LMICs to help define treatment paradigms relevant to the local African context. In a systematic review of all phase 3 oncology RCTs published globally from 2014 to 2017, Wells et al. demonstrated that although RCTs are predominantly performed in HICs, RCTs from LMICs more successfully identify effective therapies and have larger effect sizes [39]. They also showed that RCTs in HICs were more likely to be industry-funded (464 [73%] vs. 24 [41%]; *P* < 0.001) and were disproportionately focused on breast cancer compared to other cancers (e.g., cervical cancer) relative to their global cancer mortality burden [39]. This disparity likely contributes to publication and funding bias against RCTs in LMICs.

## Conclusions

Endometrial cancer research in Africa is extremely limited, with the majority being concentrated in African countries with higher HDIs. As the incidence of endometrial cancer rises in Sub-Saharan Africa, there is a pressing need for more prospective clinical research to tackle the growing disease burden and tailor treatment to each patient’s biology, local environment, and socio-politico-economic environment. Our systematic review demonstrates that the landscape of endometrial cancer research in Africa does not match the increasing burden of endometrial cancer. Moreover, the endometrial cancer data that exist globally cannot be generalized to the majority of women in sub-Saharan Africa, who tend to have more aggressive histologies, present with later stages of cancer, and lack access to all treatment modalities. This review should serve as a call to action to increase the number and quality of endometrial cancer research studies in Sub-Saharan Africa.

## Limitations

There are some limitations to our study. Stage data were not widely available in the included studies. Available data would not be helpful due to the selective nature of some of the papers (i.e., paper on select stages rather than on all stages). Most of the studies were retrospective and lacked a formal control.

### Supplementary Information

**Additional file 1. Tables S1–S7: Supplementary Table S1. **Ovid MEDLINE search strategy. **Supplementary Table S2**. Ovid Embase search strategy.** Supplementary Table S3. **Clarivate Analytics Web of Science search strategy.** Supplementary Table S4. **Wiley-Blackwell Cochrane Library search strategy.** Supplementary Table S5. **WHO African Index Medicus Database.** Supplementary Table S6. **Results of critical appraisal of included observational studies using Newcastle‒Ottawa scores.** Supplementary Table S7. **Results of critical appraisal of included randomized controlled trials using Cochrane Risk Of Bias 2.

## Data Availability

The datasets used and/or analysed during the current study are available from the corresponding author on reasonable request.
