# Supplementary material for: A systematic review of endometrial cancer clinical research in Africa
Source: Infect Agent Cancer. 2024 Jan 12;19:2. doi: 10.1186/s13027-023-00563-2 (PMC10787484; doi:10.1186/s13027-023-00563-2)
Supplement: Supplementary file 1 — Additional file 1. Tables S1–S7: Supplementary Table S1. Ovid MEDLINE search strategy. Supplementary Table S2. Ovid Embase search strategy. Supplementary Table S3. Clarivate Analytics Web of Science search strategy. Supplementary Table S4. Wiley-Blackwell Cochrane Library search strategy. Supplementary Table S5. WHO African Index Medicus Database. Supplementary Table S6. Results of critical appraisal of included observational studies using Newcastle‒Ottawa scores. Supplementary Table S7. Results of critical appraisal of included randomized controlled trials using Cochrane Risk Of Bias 2. [file 13027_2023_563_MOESM1_ESM.docx]

**Additional file 1: APPENDIX: Tables S1- S7**

**Supplementary Table S1. Ovid MEDLINE search strategy**

| # | Search |
| --- | --- |
| 1 | exp Endometrial Neoplasms/ |
| 2 | (endometri* adj5 (cancer* or neoplas* or carcinom* or adenocarcinom*)).ti, ab,kf. |
| 3 | 1 or 2 [EC: endometrial cancer] |
| 4 | exp africa/ |
| 5 | (Algeria or Angola or Benin or Botswana or "Burkina Faso" or Burundi or Cabo Verde or Cameroon or "Central African Republic" or Comoros or Congo or "Cote d'Ivoire" or Djibouti or Egypt or Guinea or Eritrea or eSwatini or Swaziland or Ethiopia or Gabon or Gambia or Ghana or "Guinea-Bissau" or Kenya or Lesotho or Liberia or Libya or Madagascar or Malawi or Mali or Mauritania or Mauritius or Morocco or Mozambique or Namibia or Niger or Nigeria or Rwanda or Sahrawi or Seychelles or "Sao Tome" or Senegal or "Sierra Leone" or Somalia or "South Africa" or Sudan or Tanzania or Togo or Tunisia or Uganda or Zambia or Zimbabwe).ti, ab,kf. |
| 6 | (Algeria or Angola or Benin or Botswana or "Burkina Faso" or Burundi or Cabo Verde or Cameroon or "Central African Republic" or Comoros or Congo or "Cote d'Ivoire" or Djibouti or Egypt or Guinea or Eritrea or eSwatini or Swaziland or Ethiopia or Gabon or Gambia or Ghana or "Guinea-Bissau" or Kenya or Lesotho or Liberia or Libya or Madagascar or Malawi or Mali or Mauritania or Mauritius or Morocco or Mozambique or Namibia or Niger or Nigeria or Rwanda or Sahrawi or Seychelles or "Sao Tome" or Senegal or "Sierra Leone" or Somalia or "South Africa" or Sudan or Tanzania or Togo or Tunisia or Uganda or Zambia or Zimbabwe).in. and (Algeria or Angola or Benin or Botswana or "Burkina Faso" or Burundi or Cabo Verde or Cameroon or "Central African Republic" or Comoros or Congo or "Cote d'Ivoire" or Djibouti or Egypt or Guinea or Eritrea or eSwatini or Swaziland or Ethiopia or Gabon or Gambia or Ghana or "Guinea-Bissau" or Kenya or Lesotho or Liberia or Libya or Madagascar or Malawi or Mali or Mauritania or Mauritius or Morocco or Mozambique or Namibia or Niger or Nigeria or Rwanda or Sahrawi or Seychelles or "Sao Tome" or Senegal or "Sierra Leone" or Somalia or "South Africa" or Sudan or Tanzania or Togo or Tunisia or Uganda or Zambia or Zimbabwe).cp |
| 7 | (Algeria or Angola or Benin or Botswana or "Burkina Faso" or Burundi or Cabo Verde or Cameroon or "Central African Republic" or Comoros or Congo or "Cote d'Ivoire" or Djibouti or Egypt or Guinea or Eritrea or eSwatini or Swaziland or Ethiopia or Gabon or Gambia or Ghana or "Guinea-Bissau" or Kenya or Lesotho or Liberia or Libya or Madagascar or Malawi or Mali or Mauritania or Mauritius or Morocco or Mozambique or Namibia or Niger or Nigeria or Rwanda or Sahrawi or Seychelles or "Sao Tome" or Senegal or "Sierra Leone" or Somalia or "South Africa" or Sudan or Tanzania or Togo or Tunisia or Uganda or Zambia or Zimbabwe).in./freq=2 |
| 8 | (africa or "africa* countr*" or Chad).ti, ab,kf. |
| 9 | ("La Reunion" adj3 France).ti, ab,kf, in. |
| 10 | or/4-9 [for studies in Africa] |
| 11 | 3 and 10 |
| 12 | (animals not (humans and animals)).sh. |
| 13 | 11 not 12 [Remove animal studies] |
| 14 | (mice or mouse or murine or rat or rats or rodent or cells or "in vitro" or "cell line").ti. |
| 15 | 13 not 14 [Further Remove animal and in vitro studies] |
| 16 | case report*.ti, jw. |
| 17 | case reports.pt. not (exp clinical study/or comparative study/or evaluation studies/or meta-analysis/or multicenter study/or validation studies/or exp Cohort Studies/or letter.pt. or (series or cohort or retrospective*).ti, ab.) |
| 18 | 16 or 17 |
| 19 | 15 not 18 |
| 20 | limit 19 to yr="2011 -Current" |

**Supplementary Table S2. Ovid Embase search strategy**

| # | Search |
| --- | --- |
| 1 | exp endometrium cancer/ |
| 2 | (endometri* adj5 (cancer* or neoplas* or carcinom* or adenocarcinom*)).ti, ab. |
| 3 | 1 or 2 |
| 4 | exp Africa/ |
| 5 | (Algeria or Angola or Benin or Botswana or "Burkina Faso" or Burundi or Cabo Verde or Cameroon or "Central African Republic" or Comoros or Congo or "Cote d'Ivoire" or Djibouti or Egypt or Guinea or Eritrea or eSwatini or Swaziland or Ethiopia or Gabon or Gambia or Ghana or "Guinea-Bissau" or Kenya or Lesotho or Liberia or Libya or Madagascar or Malawi or Mali or Mauritania or Mauritius or Morocco or Mozambique or Namibia or Niger or Nigeria or Rwanda or Sahrawi or Seychelles or "Sao Tome" or Senegal or "Sierra Leone" or Somalia or "South Africa" or Sudan or Tanzania or Togo or Tunisia or Uganda or Zambia or Zimbabwe).ti, ab,kw. |
| 6 | (Algeria or Angola or Benin or Botswana or "Burkina Faso" or Burundi or Cabo Verde or Cameroon or "Central African Republic" or Comoros or Congo or "Cote d'Ivoire" or Djibouti or Egypt or Guinea or Eritrea or eSwatini or Swaziland or Ethiopia or Gabon or Gambia or Ghana or "Guinea-Bissau" or Kenya or Lesotho or Liberia or Libya or Madagascar or Malawi or Mali or Mauritania or Mauritius or Morocco or Mozambique or Namibia or Niger or Nigeria or Rwanda or Sahrawi or Seychelles or "Sao Tome" or Senegal or "Sierra Leone" or Somalia or "South Africa" or Sudan or Tanzania or Togo or Tunisia or Uganda or Zambia or Zimbabwe).in./freq=2 |
| 7 | (Algeria or Angola or Benin or Botswana or "Burkina Faso" or Burundi or Cabo Verde or Cameroon or "Central African Republic" or Comoros or Congo or "Cote d'Ivoire" or Djibouti or Egypt or Guinea or Eritrea or eSwatini or Swaziland or Ethiopia or Gabon or Gambia or Ghana or "Guinea-Bissau" or Kenya or Lesotho or Liberia or Libya or Madagascar or Malawi or Mali or Mauritania or Mauritius or Morocco or Mozambique or Namibia or Niger or Nigeria or Rwanda or Sahrawi or Seychelles or "Sao Tome" or Senegal or "Sierra Leone" or Somalia or "South Africa" or Sudan or Tanzania or Togo or Tunisia or Uganda or Zambia or Zimbabwe).ad. |
| 8 | (Algeria or Angola or Benin or Botswana or "Burkina Faso" or Burundi or Cabo Verde or Cameroon or "Central African Republic" or Comoros or Congo or "Cote d'Ivoire" or Djibouti or Egypt or Guinea or Eritrea or eSwatini or Swaziland or Ethiopia or Gabon or Gambia or Ghana or "Guinea-Bissau" or Kenya or Lesotho or Liberia or Libya or Madagascar or Malawi or Mali or Mauritania or Mauritius or Morocco or Mozambique or Namibia or Niger or Nigeria or Rwanda or Sahrawi or Seychelles or "Sao Tome" or Senegal or "Sierra Leone" or Somalia or "South Africa" or Sudan or Tanzania or Togo or Tunisia or Uganda or Zambia or Zimbabwe).cp. and (Algeria or Angola or Benin or Botswana or "Burkina Faso" or Burundi or Cabo Verde or Cameroon or "Central African Republic" or Comoros or Congo or "Cote d'Ivoire" or Djibouti or Egypt or Guinea or Eritrea or eSwatini or Swaziland or Ethiopia or Gabon or Gambia or Ghana or "Guinea-Bissau" or Kenya or Lesotho or Liberia or Libya or Madagascar or Malawi or Mali or Mauritania or Mauritius or Morocco or Mozambique or Namibia or Niger or Nigeria or Rwanda or Sahrawi or Seychelles or "Sao Tome" or Senegal or "Sierra Leone" or Somalia or "South Africa" or Sudan or Tanzania or Togo or Tunisia or Uganda or Zambia or Zimbabwe).in. |
| 9 | (africa or "africa* countr*" or Chad).ti, ab,kw. |
| 10 | ("La Reunion" adj3 France).ti, ab,kw, in,ad. |
| 11 | or/4-10 [Africa countries] |
| 12 | 3 and 11 |
| 13 | Human/ |
| 14 | Nonhuman/or ANIMAL/or Animal Experiment/ |
| 15 | 14 not 13 |
| 16 | 12 not 15 |
| 17 | (mice or mouse or murine or rat or rats or rodent or cells or "in vitro" or "cell line").ti. |
| 18 | 16 not 17 [Further Remove animal and in vitro studies] |
| 19 | case report/or case report.ti. |
| 20 | 18 not 19 [Remove some case reports] |
| 21 | exp *endometrium cancer/ |
| 22 | exp endometrium cancer/and (endometri* adj2 (cancer* or neoplas* or carcinom* or adenocarcinom*)).ab. |
| 23 | (endometri* adj2 (cancer* or neoplas* or carcinom* or adenocarcinom*)).ti, kw. |
| 24 | (endometri* adj2 (cancer* or neoplas* or carcinom* or adenocarcinom*)).ab./freq=2 |
| 25 | or/21-24 |
| 26 | 20 and 25 |
| 27 | limit 26 to yr="2011 -Current" |

**Supplementary Table S3. Clarivate Analytics Web of Science search strategy**

| # | Search |
| --- | --- |
| 19 | #18  AND  #14 |
| 18 | #17  OR  #16  OR  #15 |
| 17 | AK=  ((endometri* OR uterine)  NEAR/2  (cancer* or neoplas* or carcinom* or adenocarcinom*) ) |
| 16 | AB=  ((endometri* OR uterine)  NEAR/2  (cancer* or neoplas* or carcinom* or adenocarcinom*)  ) |
| 15 | TI=  (endometri* or uterine) |
| 14 | #12  NOT  #13 |
| 13 | TITLE:  (case report*) |
| 12 | #10  not  #11 |
| 11 | TITLE:  ((mice or mouse or murine or rat or rats or rodent or cells or "in vitro" or "cell line") ) |
| 10 | #9  AND  #1 |
| 9 | #8  OR  #7  OR  #6  OR  #5  OR  #4  OR  #3  OR  #2 |
| 8 | AD=("La  Reunion"  NEAR/3  France) |
| 7 | OO=("La  Reunion"  NEAR/3  France) |
| 6 | TOPIC:  (("La Reunion" NEAR/3 France) ) |
| 5 | TOPIC:  ((africa or "african countr*" or Chad) ) |
| 4 | OO=(Algeria  or  Angola  or  Benin  or  Botswana  or  "Burkina  Faso"  or  Burundi  or  Cabo  Verde  or  Cameroon  or  "Central  African  Republic"  or  Comoros  or  Congo  or  "Cote  d'Ivoire"  or  Djibouti  or  Egypt  or  Guinea  or  Eritrea  or  eSwatini  or  Swaziland  or  Ethiopia  or  Gabon  or  Gambia  or  Ghana  or  "Guinea-Bissau"  or  Kenya  or  Lesotho  or  Liberia  or  Libya  or  Madagascar  or  Malawi  or  Mali  or  Mauritania  or  Mauritius  or  Morocco  or  Mozambique  or  Namibia  or  Niger  or  Nigeria  or  Rwanda  or Sahrawi or Seychelles or  "Sao  Tome"  or  Senegal  or  "Sierra  Leone"  or  Somalia  or  "South  Africa"  or  Sudan  or  Tanzania  or  Togo  or  Tunisia  or  Uganda  or  Zambia  or  Zimbabwe) |
| 3 | AD=(Algeria  or  Angola  or  Benin  or  Botswana  or  "Burkina  Faso"  or  Burundi  or  Cabo  Verde  or  Cameroon  or  "Central  African  Republic"  or  Comoros  or  Congo  or  "Cote  d'Ivoire"  or  Djibouti  or  Egypt  or  Guinea  or  Eritrea  or  eSwatini  or  Swaziland  or  Ethiopia  or  Gabon  or  Gambia  or  Ghana  or  "Guinea-Bissau"  or  Kenya  or  Lesotho  or  Liberia  or  Libya  or  Madagascar  or  Malawi  or  Mali  or  Mauritania  or  Mauritius  or  Morocco  or  Mozambique  or  Namibia  or  Niger  or  Nigeria  or  Rwanda  or Sahrawi or Seychelles or  "Sao  Tome"  or  Senegal  or  "Sierra  Leone"  or  Somalia  or  "South  Africa"  or  Sudan  or  Tanzania  or  Togo  or  Tunisia  or  Uganda  or  Zambia  or  Zimbabwe) |
| 2 | TOPIC:  ((Algeria or Angola or Benin or Botswana or "Burkina Faso" or Burundi or Cabo Verde or Cameroon or "Central African Republic" or Comoros or Congo or "Cote d'Ivoire" or Djibouti or Egypt or Guinea or Eritrea or eSwatini or Swaziland or Ethiopia or Gabon or Gambia or Ghana or "Guinea-Bissau" or Kenya or Lesotho or Liberia or Libya or Madagascar or Malawi or Mali or Mauritania or Mauritius or Morocco or Mozambique or Namibia or Niger or Nigeria or Rwanda or Sahrawi or Seychelles or "Sao Tome" or Senegal or "Sierra Leone" or Somalia or "South Africa" or Sudan or Tanzania or Togo or Tunisia or Uganda or Zambia or Zimbabwe) ) |
| 1 | TS=((endometri*  NEAR/4  (cancer* or neoplas* or carcinom* or adenocarcinom*) ))  Indexes=SCI-EXPANDED, SSCI, A&HCI, CPCI-S, CPCI-SSH, ESCI Timespan=2011-2021 |

**Supplementary Table S4. Wiley-Blackwell Cochrane Library search strategy**

| # | Search |
| --- | --- |
| 1 | MeSH descriptor: [Endometrial Neoplasms] explode all trees |
| 2 | ((endometri* NEAR/5 (cancer* or neoplas* or carcinom* or adenocarcinom*))):ti, ab,kw (Word variations have been searched) |
| 3 | #1 OR #2 |
| 4 | MeSH descriptor: [Africa] explode all trees |
| 5 | ((Algeria or Angola or Benin or Botswana or "Burkina Faso" or Burundi or Cabo Verde or Cameroon or "Central African Republic" or Comoros or Congo or "Cote d'Ivoire" or Djibouti or Egypt or Guinea or Eritrea or eSwatini or Swaziland or Ethiopia or Gabon or Gambia or Ghana or "Guinea-Bissau" or Kenya or Lesotho or Liberia or Libya or Madagascar or Malawi or Mali or Mauritania or Mauritius or Morocco or Mozambique or Namibia or Niger or Nigeria or Rwanda or Sahrawi or Seychelles or "Sao Tome" or Senegal or "Sierra Leone" or Somalia or "South Africa" or Sudan or Tanzania or Togo or Tunisia or Uganda or Zambia or Zimbabwe)) (Word variations have been searched) |
| 6 | ((africa or "africa* countr*" or Chad)):ti, ab,kw (Word variations have been searched) |
| 7 | (("La Reunion" NEAR/3 France)) (Word variations have been searched) |
| 8 | #4 OR #5 OR #6 OR #7 |
| 9 | #3 AND #8 |

**Supplementary Table S5. WHO African Index Medicus Database**

| URL | Search terms |
| --- | --- |
| https://www.globalindexmedicus.net/ | "endometrial cancer" |
| https://indexmedicus.afro.who.int/ | “endometrial"; "endometriod", "endomètre" "endometrium" |

**Supplementary Table S6.** Results of critical appraisal of included observational studies using Newcastle‒Ottawa scores.

| **Study** | **Study Design** | **Selection** | | | | **Comparability** | **Outcome** | | | **Total**  **Score** |
| --- | --- | --- | --- | --- | --- | --- | --- | --- | --- | --- |
|  |  | Case Definition Adequate? | Case Representative? | Selection of Controls | Definition of Controls | Based on Design or Analysis | Ascertainment of Exposure | Same method for Cases and Controls | Nonresponse Rate |  |
| Ray, 2019 | Case Control | + | + | - | + | + | + | + | + | **7,**  **Good** |

| **Study** | **Study Design** | **Selection** | | | | **Comparability** | **Outcome** | | | **Total Score** |
| --- | --- | --- | --- | --- | --- | --- | --- | --- | --- | --- |
|  |  | Exposed cohort Representative? | Selection of Non Exposed Cohort | Ascertainment of Exposure | Outcome not present at study start? | Based on Design or Analysis | Assessment of Outcome | Timing of Follow-up | Adequate Follow-Up |  |
| Ghazala,  2021 | Cohort | + | - | + | - | ++ | + | + | + | **7, Fair** |
| Abouhashem, 2016 | Cohort | + | + | + | + | ++ | + | + | - | **8,**  **Good** |
| Aly,  2013 | Cohort | + | + | + | + | ++ | + | + | + | **9,**  **Good** |
| El Sokkary,  2014 | Cohort | + | + | + | + | + | + | + | + | **8,**  **Good** |
| Gharib,  2020 | Cohort | + | + | + | + | + | + | + | - | **7,**  **Good** |
| Hamed,  2012 | Cohort | + | + | + | + | + | + | + | + | **8,**  **Good** |
| Mourad,  2017 | Cohort | + | + | + | + | ++ | + | + | + | **9, Good** |
| Sanad,  2019 | Cohort | + | + | + | + | **-^i^** | + | + | + | **7, Fair ^i^** |
| Shady,  2016 | Cohort | + | + | + | + | ++ | + | + | + | **9, Good** |
| Shatat,  2019 | Cohort | + | + | + | + | **-^i^** | + | + | + | **7, Fair ^i^** |
| Soliman,  2011 | Cohort | + | + | + | + | **-^i^** | + | + | + | **7, Fair** **^i^** |
| Rady,  2019 **^ii^** | Nonrandomized experimental study | + | + | + | + | + | + | + | + | **8,**  **Good** |

^i^ These studies had an inadequate degree of control; thus, the total score was based on this.

^ii^ This nonrandomized experimental study was evaluated as a cohort study.

| **Study** | **Study Design** | **Selection** | | | | **Comparability** | **Outcome** | | **Total**  **Score** |
| --- | --- | --- | --- | --- | --- | --- | --- | --- | --- |
|  |  | Representative Sample? | Sample Size Adequate | Non Respondents | Ascertainment of Exposure | Based on Design or Analysis | Assessment of Outcome | Statistical Test |  |
| Wadee,  2021 **^iii^** | Cross- sectional | - | - | + | ++ | ++ | ++ | + | **8,**  **Good** |
| Elmahdy,  2019 | Cross- sectional | + | - | + | + | + | ++ | + | **7,**  **Good** |
| Ghorbel,  2020 | Cross- sectional | + | - | - | ++ | ++ | + | + | **7,**  **Good** |

^iii^ This author utilized the same patient population for 2 studies, alternately describing the design as a cohort vs a cross-sectional/diagnostic accuracy study. The cohort study was excluded.

**Supplementary Table S7.** Results of critical appraisal of included randomized controlled trials using Cochrane Risk Of Bias 2

| **Study** | **Study Design** | **Sequence Generation** | **Allocation Concealment** | **Participant and Personnel Blinding** | **Outcome Assessment Blinding** | **Incomplete Outcome Data** | **Selective Reporting** | **Other Bias Sources** | **Total**  **Bias Risk** |
| --- | --- | --- | --- | --- | --- | --- | --- | --- | --- |
| El-Agwany, 2018 | Randomized control | High | High | High | Unclear | Unclear | Low | Unclear | **High** |
| Fayallah,  2011 | Randomized control | Low | Low | Low | Unclear | Low | Unclear | Unclear | **Some concern** |
